# *Culex pipiens* and *Aedes triseriatus* Mosquito Susceptibility to Zika Virus

**DOI:** 10.3201/eid2210.161082

**Published:** 2016-10

**Authors:** Matthew T. Aliota, Stephen A. Peinado, Jorge E. Osorio, Lyric C. Bartholomay

**Affiliations:** University of Wisconsin, Madison, Wisconsin, USA

**Keywords:** Zika virus, vector competence, *Aedes aegypti*, *Aedes albopictus*, *Aedes triseriatus*, *Culex pipiens*, vector-borne infections, mosquito, Flavivirus, *Flaviviridae*, viruses

**To the Editor:** Zika virus, genus *Flavivirus*, has spread nearly uncontrolled since its introduction into the Western Hemisphere; autochthonous spread has occurred in >39 countries and territories, including several US territories. Transmission of Zika virus is usually by the bite of infected mosquitoes, and potential for emergence in areas with competent mosquito vectors is high (*1*). Future spread of Zika virus is unpredictable; however, eventual local spread in the United States is possible. As of July 13, 2016, a total of 1,306 travel-associated cases had been reported (ArboNET, https://www.cdc.gov/zika); substantial populations of *Aedes (Stegomyia) aegypti* (Linnaeus) mosquitoes exist in >16 states in the eastern, southeastern, and southwestern United States; and *Ae. (Stegomyia) albopictus* (Skuse) mosquitoes inhabit >28 states and continued expansion throughout the northern United States is probable (*2*). Mosquitoes of these 2 species have demonstrated the ability to transmit Zika virus (*1*).

The recent epidemic spread of Zika virus suggests that *Ae. aegypti* mosquitoes are the main vector; however, information about the role of other species in driving and maintaining Zika virus transmission is lacking. Of particular concern this summer (2016) is emergence and establishment of Zika virus in previously unaffected geographic areas; with the advent of mosquito season commencing in most of the continental United States, the likelihood of mosquitoborne transmission of Zika virus in states without populations of *Ae. aegypti* and *Ae. albopictus* mosquitoes remains unknown. To understand the potential risk for spread of Zika virus in temperate US states, we compared the relative abilities of *Culex pipiens* and *Ae. triseriatus* mosquitoes to transmit Zika virus in the laboratory. We used *Ae. aegypti* and *Ae. albopictus* mosquitoes as positive controls.

Laboratory colonies of mosquitoes used in this study were maintained at the University of Wisconsin–Madison, and vector competence for Zika virus was evaluated by using established procedures (*3*,*4*). Mosquitoes from each group were incapacitated (exposed to trimethylamine); legs were removed and collected. Salivary secretions were collected in capillary tubes containing a 1:1 ratio of fetal bovine serum and 50% sucrose. Mosquitoes were then placed in individual tubes; their bodies and legs were homogenized, clarified by centrifugation, and screened for virus infection. Dissemination was indicated by virus-positive legs, and transmission potential was indicated by virus-positive salivary secretions. All samples were screened by plaque assay on Vero cells. Mosquitoes were exposed to Asian lineage Zika virus strain PRVABC59 (GenBank accession no. KU501215) (*5*) by feeding on Zika virus-infected *Ifnar−/−* mice (*4*). Mice (n = 4/replicate) yielded infectious blood meal concentrations of 6.02 log_10_ PFU/mL ± 0.67 (mean ± SD; biological replicate no. 1), 4.74 log_10_ PFU/mL ± 0.06 (replicate no. 2), and 6.83 log_10_ PFU/mL ± 0.45 (replicate no. 3). Blood meal concentrations in mice were consistent with viremia concentrations of humans in the field (*4*).

All samples from *Cx. pipiens* mosquitoes and all replicates were negative for Zika virus by plaque assay (Table). In contrast, *Ae. triseriatus* mosquitoes were susceptible to infection when exposed to mice with the highest viremia concentrations (Table). However, none of these infected mosquitoes disseminated virus and none were capable of transmitting the virus. Data from *Ae. albopictus* and *Ae. aegypti* mosquitoes that had been exposed to the same mice demonstrated that the viremia concentrations used could productively infect mosquitoes. Of note, *Ae. albopictus* mosquito infection rates were dose dependent (i.e., infection rates increased with blood meal titer). Furthermore, data generated from exposure to the same mice demonstrated productive mosquito infection with these viremia concentrations (*4*). It therefore seems likely that if Zika virus circulation in the United States occurs, it will be driven by *Ae. albopictus* or *Ae. aegypti* mosquitoes (*6*). However, we cannot rule out that anthropophilic mosquitoes of other species in this country could be competent vectors.

**Table:** Competence of mosquitoes, by species, as Zika virus vectors, 14 days after peroral infection, United States*

Mosquito species	No. virus-positive/no. tested (%)										
	Biological replicate 1, mean 6.02 log_10_ PFU/mL ± SD 0.67				Biological replicate 2, mean 4.74 log_10_ PFU/mL ± SD 0.06				Biological replicate 3, mean 6.83 log_10_ PFU/mL ± SD 0.45		
	I	D	T		I	D	T		I	D	T
*Culex pipiens*†	0/20 (0)	0/20 (0)	0/20 (0)		0/10 (0)	0/10 (0)	0/10 (0)		0/30 (0)	0/30 (0)	0/30 (0)
*Aedes triseriatus*‡	ND	ND	ND		0/20 (0)	0/20 (0)	0/20 (0)		4/13 (31)	0/4 (0)	0/4 (0)
*Ae. albopictus*§	9/9 (100)	6/9 (67)	2/9 (22)		1/6 (17)	0/1 (0)	0/1 (0)		ND	ND	ND
*Ae. aegypti*¶	ND	ND	ND		ND	ND	ND		17/17 (100)	12/17 (71)	4/17 (24)

*Zika virus strain PRVABC59 (GenBank accession no.KU501215) was originally isolated from a traveler to Puerto Rico in December 2015. I, infected; D, disseminated; ND, no data; T, transmitted. †Originated from egg rafts collected in Iowa in 2002 and colonized at the Iowa State University Medical Entomology Laboratory. ‡Originated from eggs collected in Iowa in 2002 and 2003 and colonized at the Iowa State University Medical Entomology Laboratory. §Originated from eggs collected in Missouri in 2002 and colonized at the Illinois Natural History Survey. *¶*Black-eyed Liverpool strain.

These data argue for continued studies (experimental and epidemiologic) assessing interactions between differing mosquito–Zika virus combinations in the United States because of geographic variations that may exist in oral susceptibility of mosquitoes of the same or different species. The few vector competence studies conducted to date have focused primarily on *Ae. aegypti* and *Ae. albopictus* mosquitoes (*8*), but mosquitoes of other species may be vectors, depending on geographic location. We focused on *Cx. pipiens* mosquitoes because they are ubiquitous (*7*), they are considered one of the principal vectors of West Nile virus in the northern half of the United States, and a recent report from Brazil suggests *Cx. quinquefasciatus* mosquitoes as potential Zika virus vectors (*8*). We chose *Ae. triseriatus* mosquitoes because they are the natural vector and overwintering host of La Crosse virus, they are extremely tolerant to a range of temperatures, they are distributed from Florida to eastern Canada (*9*), and they have been implicated as potential enzootic vectors for West Nile virus (*10*). To determine the risk for Zika virus transmission in the United States, surveillance of different human-biting mosquito species will be paramount. Although we expected that *Cx. pipiens* and *Ae. triseriatus* mosquitoes would not be competent Zika virus vectors, our experimental verification helps exclude uncertainties surrounding the potential vectors of this emerging pathogen.
